# Supplementary material for: Predicting visual function by interpreting a neuronal wiring diagram
Source: Nature. 2024 Oct 2;634(8032):113–23. doi: 10.1038/s41586-024-07953-5 (PMC11446822; doi:10.1038/s41586-024-07953-5)
Supplement: Supplementary file 6 — CRF and ERF predictions for individual TmY4 and TmY9 cells. Analogous to Supplementary Data 3, but for TmY target types. Shown are the top four monosynaptic pathways, the strongest pathway passing through each of the top ten intermediary types (ranking from Extended Data Fig. 7), and the trisynaptic pathway Tm1–TmY–Dm3–TmY (see the section entitled Prediction of spatial normalization). [file 41586_2024_7953_MOESM6_ESM.zip › DataS4/TmY9qΓèÑ/720575940630519265.pdf]

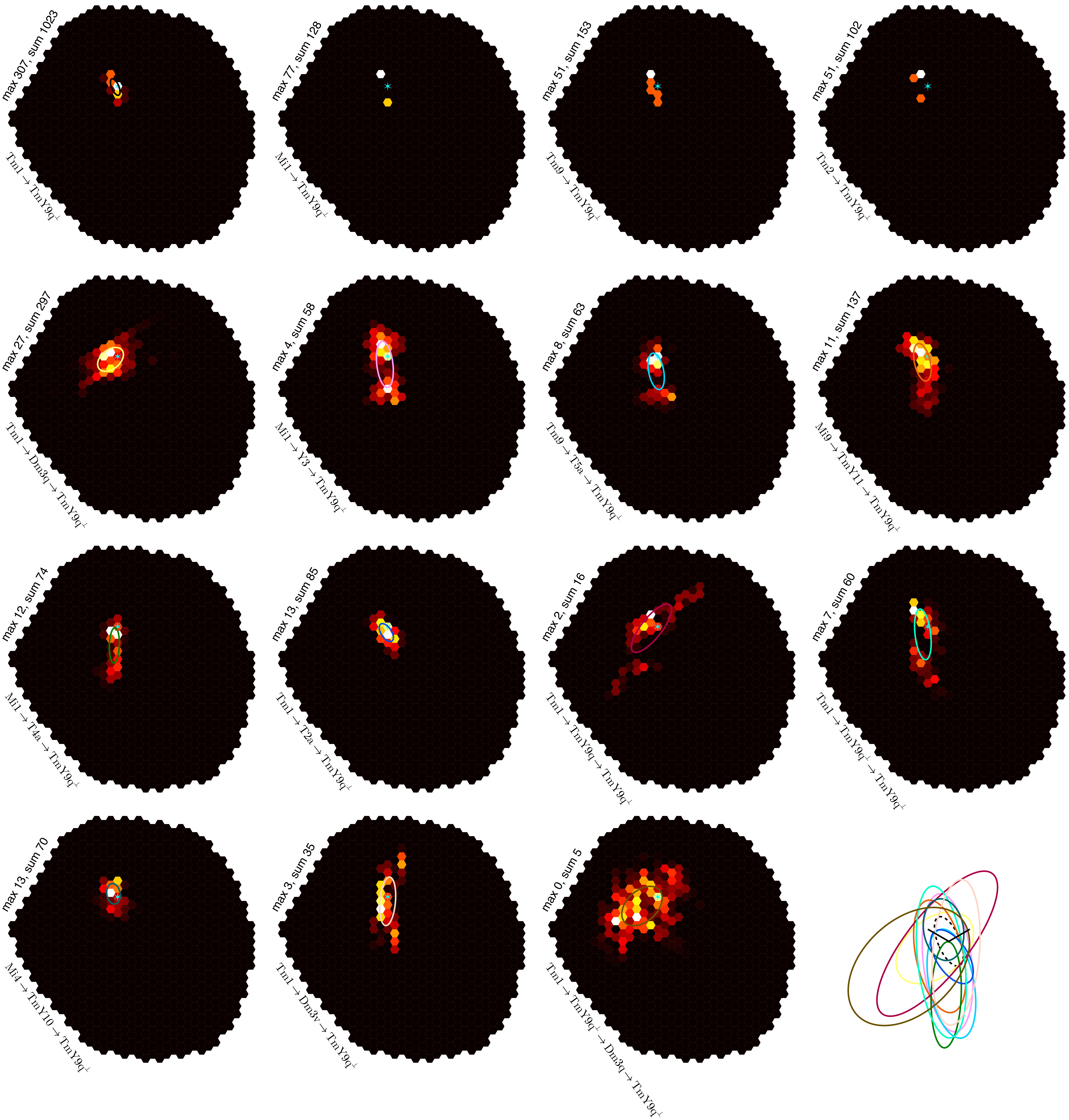
max 307, sum 1023
Tm1 → TmY9q⊥
max 77, sum 128
Mi1 → TmY9q⊥
max 51, sum 153
Tm9 → TmY9q⊥
max 51, sum 102
Tm2 → TmY9q⊥
max 27, sum 297
Tm1 → Dm3q → TmY9q⊥
max 4, sum 58
Mi1 → Y3 → TmY9q⊥
max 8, sum 63
Tm9 → T5a → TmY9q⊥
max 11, sum 137
Mi9 → TmY11 → TmY9q⊥
max 12, sum 74
Mi1 → T4a → TmY9q⊥
max 13, sum 85
Tm1 → T2a → TmY9q⊥
max 2, sum 16
Tm1 → TmY9q⊥ → TmY9q⊥
max 7, sum 60
Tm1 → TmY9q⊥ → TmY9q⊥
max 13, sum 70
Mi4 → TmY10 → TmY9q⊥
max 3, sum 35
Tm1 → Dm3v → TmY9q⊥
max 0, sum 5
Tm1 → TmY9q⊥ → Dm3q → TmY9q⊥
